# The effect of pregabalin on tourniquet-induced ischemia-reperfusion injury:a prospective randomized study

**DOI:** 10.3906/sag-1902-230

**Published:** 2019-12-16

**Authors:** Ömer KARACA, Hüseyin Ulaş PINAR, Ahmet Fevzi ÖZGÜR, Sevsen KULAKSIZOĞLU, Rafi DOĞAN

**Affiliations:** 1 Department of Anesthesiology and Reanimation, Private Anıt Hospital, Konya Turkey; 2 Department of Anesthesiology and Reanimation, School of Medicine, Başkent University, Konya Turkey; 3 Department of Orthopedics and Traumatology, School of Medicine, Başkent University, Konya Turkey; 4 Department of Biochemistry, School of Medicine, Başkent University, Konya Turkey; 5 Department of Anesthesiology and Reanimation, Çanakkale State Hospital, Çanakkale Turkey

**Keywords:** Pregabalin, antioxidant, muscle, ischemia reperfusion injury, total knee replacement

## Abstract

**Background/aim:**

The aim of this study was to investigate the efficacy of pregabalin on ischemia-reperfusion injuries.

**Materials and methods:**

Fifty-four patients were randomly assigned into 2 groups. A 150-mg tablet of pregabalin was given the night before and then 1 h before the operation for patients in Group P (pregabalin group, n = 27). A placebo was given to patients in Group C (control group, n = 27) at the same times. After combined spinal-epidural anesthesia was performed, venous blood samples were taken before tourniquet inflation (t_1_), just before tourniquet deflation (t_2_), and 20 min after tourniquet deflation (t_3_) for the analysis of total antioxidant status (TAS), total oxidant status (TOS), catalase (CAT), and ischemia-modified albumin (IMA).

**Results:**

There was no significant difference in TAS levels between the groups for the t_3_ period. However, the TAS in Group P was significantly higher in the t_3_ period than the t_2_ period (mean ± SD, 0.46 ± 0.1 vs. 0.38 ± 0.2 mmol of Trolox equivalent/L, respectively; P < 0.05). The CAT level in the t_3_ period was significantly higher in Group P than Group C (mean ± SD, 53.04 ± 32.1 vs. 35.46 ± 17.2 µmol/formaldehyde, respectively; P < 0.05). In the t_3_ period, the TOS was significantly lower in Group P than Group C (mean ± SD, 11.97 ± 5 vs. 18.29 ± 9.9 pg/mL, respectively; P < 0.05). The TOS in Group P was significantly lower in the t_3_ period than the t_2_ period (mean ± SD, 11.97 ± 5 vs. 18.98 ± 10.7 pg/mL, respectively; P < 0.0001).

**Conclusion:**

Pregabalin has no marked antioxidant activity, but it contributes to the antioxidant defense system of an organism.

## 1. Introduction

Tourniquet application during limb surgery involving stopping the blood flow (ischemia) and flow restoration (reperfusion) causes ischemia-reperfusion injury (IRI) [1]. A shortage of oxygen and nutrient supply during ischemia leads to cellular damage, which further induces the generation of reactive oxygen species (ROS); this in turn results in the modification of macromolecules such as lipids, proteins, and nucleic acids and in the alteration of functions of the aforementioned molecules in the oxygen reperfusion phase, required for cell survival [2,3]. IRI not only triggers irregularity in the cell signals responsible for ROS generation, apoptosis, and cell differentiation and proliferation, but also causes local tissue and distant organ damage owing to the release of metabolites and active neutrophils [3–5]. In human models, orthopedic surgery involving tourniquet use is a good strategy for inducing IRI [6,7].

Pregabalin is a gamma-aminobutyric acid (GABA) analogue that does not interact with GABA receptors. It reduces Ca++ influx by binding to the α2δ subunit of voltage-gated calcium channels and consequently inhibits the release of neurotransmitters such as glutamate and noradrenaline [8]. However, the mechanism of the action of pregabalin is not yet completely understood, and it is currently indicated for the treatment of diabetic neuropathy, fibromyalgia, and anxiety. Recently, it was found to be effective against postoperative pain [4,9,10]. Additionally, some experimental studies have demonstrated that pregabalin has neuroprotective, antiinflammatory, and apoptosis-reducing effects, and it was effective for improving myelin production, cerebral ischemia, and reperfusion injury [11–14]. However, no randomized clinical trials in the literature have reported the effect of pregabalin on ischemia-reperfusion. Therefore, to the best of our knowledge, ours is the first study investigating this mechanism.

In this study, we aimed to determine whether pregabalin protects against muscular ischemia-reperfusion in patients undergoing total knee replacement. The primary outcome of our study was to compare the measurements of total antioxidant status (TAS), which is a measure of the combined activities of all antioxidants, immediately before tourniquet release (ischemia, t_2_) and 20 min after tourniquet release (reperfusion, t_3_). The secondary outcome was to compare the measurements of TAS, total oxidant status (TOS), ischemia-modified albumin (IMA) level, and catalase (CAT) level before ischemia onset (t_1_) and at the t_2_ and t_3_ time points.

## 2. Materials and methods

Our study was conducted at the Başkent University Konya Application and Research Hospital after obtaining ethical approval from the Ethics Committee of Başkent University (KA 16/10). The study was registered in the Clinical Trials Registry (NCT03482544). ASA I–II patients who were aged between 18 and 65 years and had undergone unilateral total knee arthroplasty under combined spinal-epidural anesthesia were included in the study, and all the patients were informed both verbally and in writing about the details of the study before surgery.

The following patients were excluded from the study: those with a history of allergy to antiepileptic drugs and prescribed drugs; an ASA status of at least grade III; severe liver, kidney (with liver enzyme levels 3 times greater than normal levels and creatinine level greater than 1.5 mg/kg), or gastrointestinal diseases; psychiatric disorders; pregnancy or lactation; long-term opioid or nonsteroidal antiinflammatory drug use or smoking; diabetes; and other neuropathic diseases. Patients who did not provide consent to regional anesthesia and those who refused to participate were also excluded.

The patients’ ages, body mass index (BMI), sex, and ASA status were recorded*. *The patients were randomly assigned by a computerized process into 2 groups, each of which included 27 patients. Group P (experimental group) received pregabalin preoperatively, whereas Group C (control group) did not. Tablets of 150 mg of oral pregabalin (Lyrica, Pfizer, Germany) were given to patients in Group P both the evening before and 1 h before the surgery. Simultaneously, a placebo drug of the same color and size was given to participants in Group C. Electrocardiography (ECG), oxygen saturation (SpO_2_), and noninvasive blood pressure measurements were taken for patients in both groups after they were transferred to the operating room, and these measurements were recorded throughout the surgery. A 20-gauge intravenous catheter was inserted at the back of the hand to administer 0.9% NaCl (6 mL/kg), but no anxiolytic agent was administered. The first blood sample (preischemia, t_1_) was taken from the contralateral arm after performing radial arterial catheterization. The patients were then placed in a sitting position. After the skin was sterilized using an antiseptic solution, local anesthesia was administered. A 20-G Tuohy needle was inserted into the epidural space either through the L3–4 or L4–5 space using the loss of resistance technique, the subarachnoid space was accessed via the needle-through-needle technique by employing a 27-G Quincke needle, and heavy bupivacaine 0.5% (3 mL) was administered. A catheter was inserted into the epidural space for postoperative analgesia. After anesthesia administration, the patients were placed in the supine position. Surgery was initiated when the pin-prick test confirmed that the sensorial block had reached an optimal level. The tourniquet was inflated to a pressure that was 2 times more than the systolic arterial blood pressure. For conducting IMA, TAS, TOS, and CAT analyses, blood samples were processed immediately before tourniquet deflation, after the completion of surgery (ischemia, t_2_), and at 20 min after tourniquet deflation (reperfusion, t_3_). 

**Table:** Demographic characteristic of patients.

	Group C (n = 27)	Group P (n = 27)	P
Age, years	43.66 ± 11.1	46.76 ± 10.7	0.284a
BMI, kg m^–2^ ASA status (I/II)	27 ± 2.3 21/6	27.54 ± 3.6 19/8	0.518^a^ 0.535^b^
Sex (M/F)	11/16	9/18	0.573b
Duration of surgery, min	121.5 ± 14.9	117.8 ± 14.1	0.350a
Tourniquet time, min	124.4 ± 14.9	120.5 ± 14.4	0.340a

Values are presented as number or mean ± standard deviation. ASA: American Society of Anesthesiologists. BMI: Body mass index. ^a^Independent sample t-test. ^b^Chi-square test.

All blood samples were centrifuged at 3000 rpm for 10 min as soon as they were delivered to the laboratory. The serum samples were stored at –80 °C until biochemical analysis. The serum TOS levels were measured using a TAS kit (Cayman Chemical Co., Ann Arbor, MI, USA) and expressed as mmol Trolox equivalent/L. The CAT levels (µM/formaldehyde) were determined using a catalase assay kit (Cayman Chemical Co.). The serum TOS and IMA concentrations were determined using an enzyme-linked immunosorbent assay (ELISA) by employing kits purchased from Sunredbio (Shanghai, China). The serum TOS and IMA levels were expressed as pg/mL and ng/mL, respectively.

Mean arterial pressure, SpO_2_, and hazard ratio during the perioperative period, as well as side effects of pregabalin such as confusion, dry mouth, and double or blurred vision, were recorded during the perioperative and postoperative periods. Additionally, biochemical analysis results, operative time, and tourniquet time were recorded.

### 2.1. Statistical analyses

The TAS value during the reperfusion period (t_3_) was considered as the primary outcome of this study. In our preliminary study, the mean TAS values in Groups C and P were 0.377 mmol/L and 0.478 mmol/L, respectively, with a standard deviation (SD) of 0.15. According to the calculator available at http://powerandsamplesize.com, we calculated that 26 patients would be required in each group based on a power of 80% and an alpha error of 0.05. Therefore, assuming a drop-out rate of 5%, a total sample size of 54 subjects (27 subjects per group) was planned.

The analyses were conducted using SAS University 9.4 software. The continuous variables were represented as means and SDs, whereas the categorical variables were represented as frequencies and percentages. A chi-square test was used for comparing categorical variables and a t-test was used for comparing continuous variables. Mixed-effects models were developed to evaluate the effects of pregabalin and time on continuous outcomes. Inter- and intragroup comparisons of least squares mean values were performed using Tukey–Kramer adjustments whenever applicable. P < 0.05 was considered to be statistically significant.

## 3. Results

**Figure 1:**
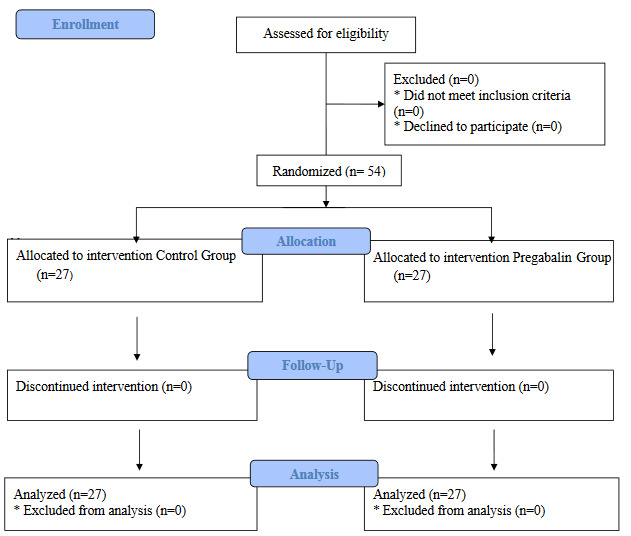
CONSORT flow diagram of the present study.

The patients eligible for this study were analyzed in terms of their primary outcomes, and the results were presented in a Consolidated Standards of Reporting Trials (CONSORT) flow diagram (Figure 1)*.*

The groups were comparable with respect to age, BMI, sex, ASA status, operative time, and tourniquet time (Table).

**Figure 2:**
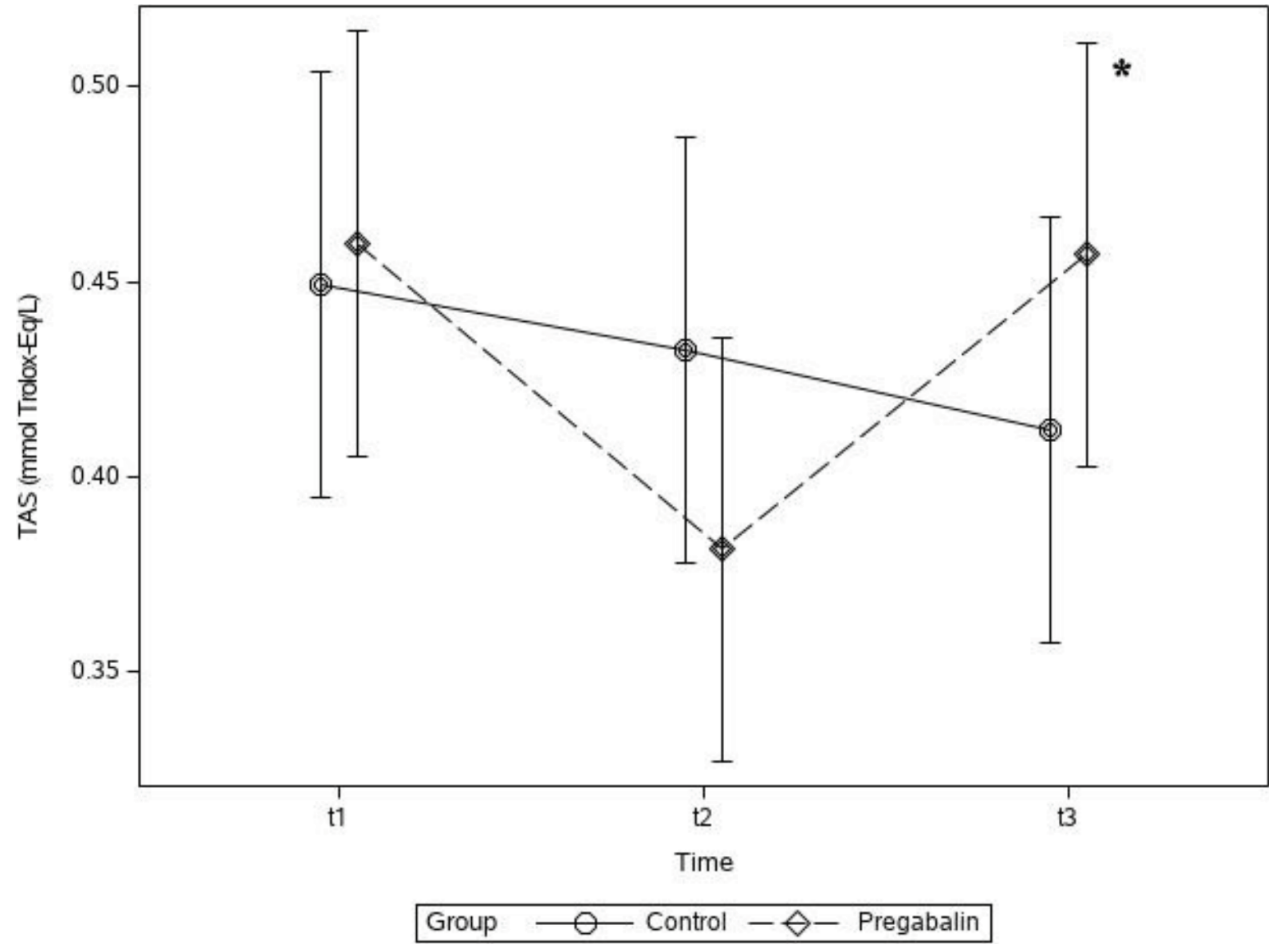
Plasma total antioxidant status (TAS) concentration levels of groups. * P < 0.05 between t_3_ and t_2_ periods in Group P.

The TAS level was high in Group P and low in Group C in the t_3_ period; however, the difference in this value between the 2 groups was not statistically significant (P > 0.05). In Group P, the TAS level was higher during the t_3_ period than during the t_2_ period, and this difference was statistically significant (mean ± SD, 0.46 ± 0.1 vs. 0.38 ± 0.2 mmol of Trolox equivalent/L; P < 0.05) (Figure 2).

**Figure 3:**
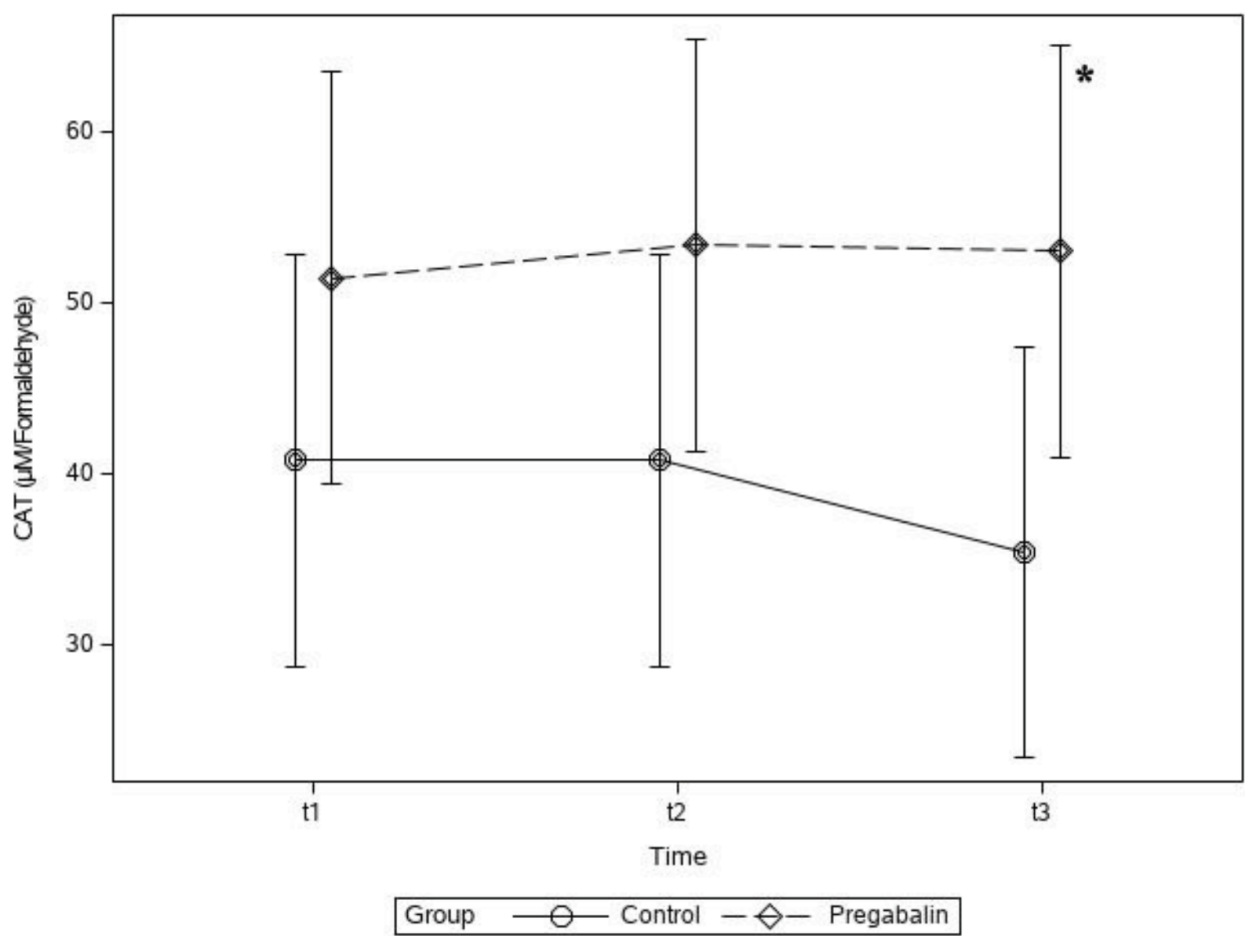
Plasma catalase (CAT) concentration levels of groups. * P < 0.05 between groups in the t_3_ period.

In the t_3_ period, the CAT level in Group P was significantly higher than that in Group C (mean ± SD, 53.04 ± 32.1 vs. 35.46 ± 17.2 µmol/formaldehyde; P < 0.05) (Figure 3). 

**Figure 4:**
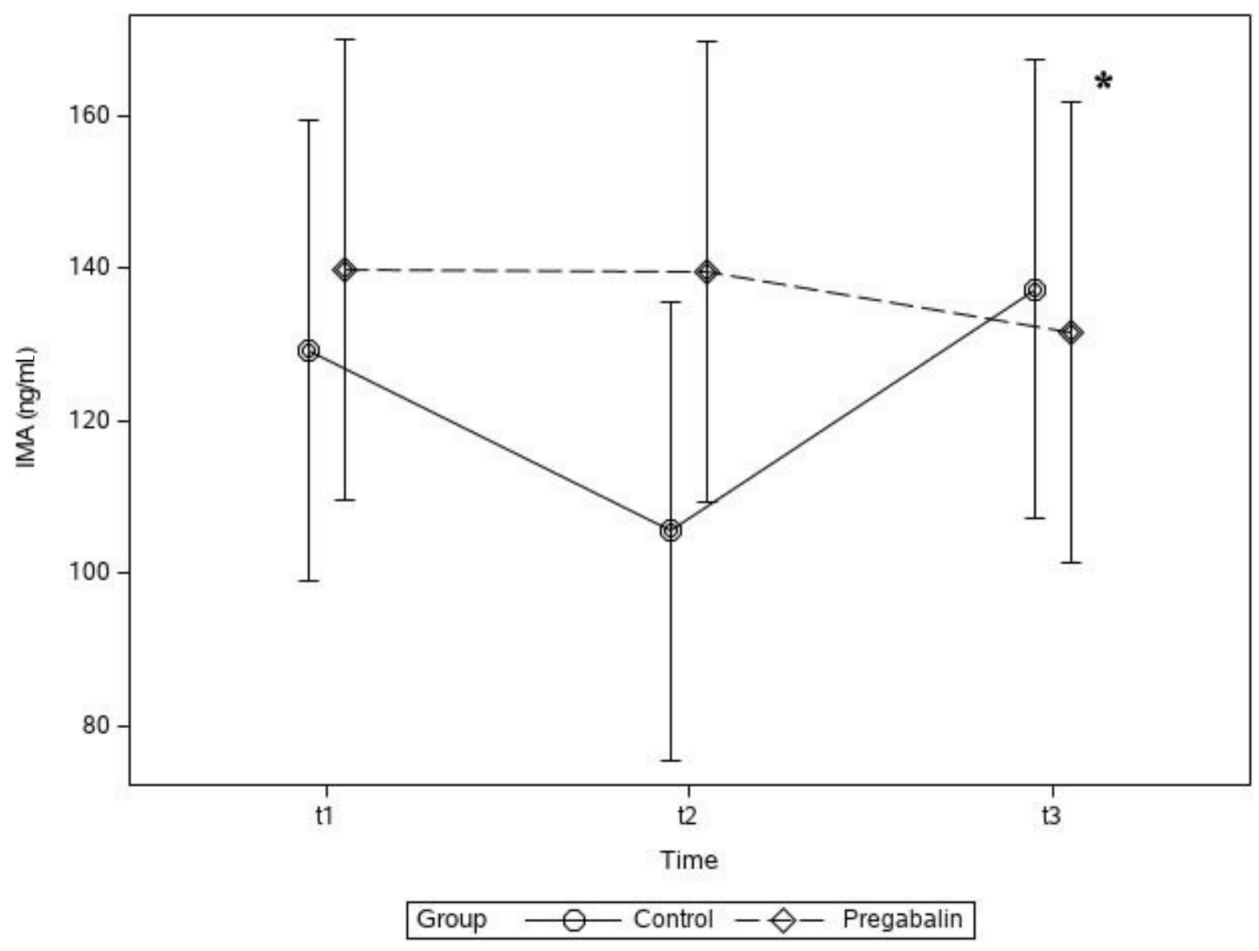
Plasma ischemia-modified albumin (IMA) concentration levels of groups. * P < 0.05 between t_3_ and t_2_ periods in Group C.

In the t_1_, t_2_, and t_3_ periods, there was no statistically significant difference between the groups in terms of IMA; however, the group × time interaction was statistically significant (P < 0.05). In the control group, the IMA value was higher in the t_3_ period than that in the t_2_ period, and this difference was statistically significant (mean ± SD, 137.21 ± 71.2 vs. 105.50 ± 54.4 ng/mL; P < 0.05) (Figure 4). 

**Figure 5:**
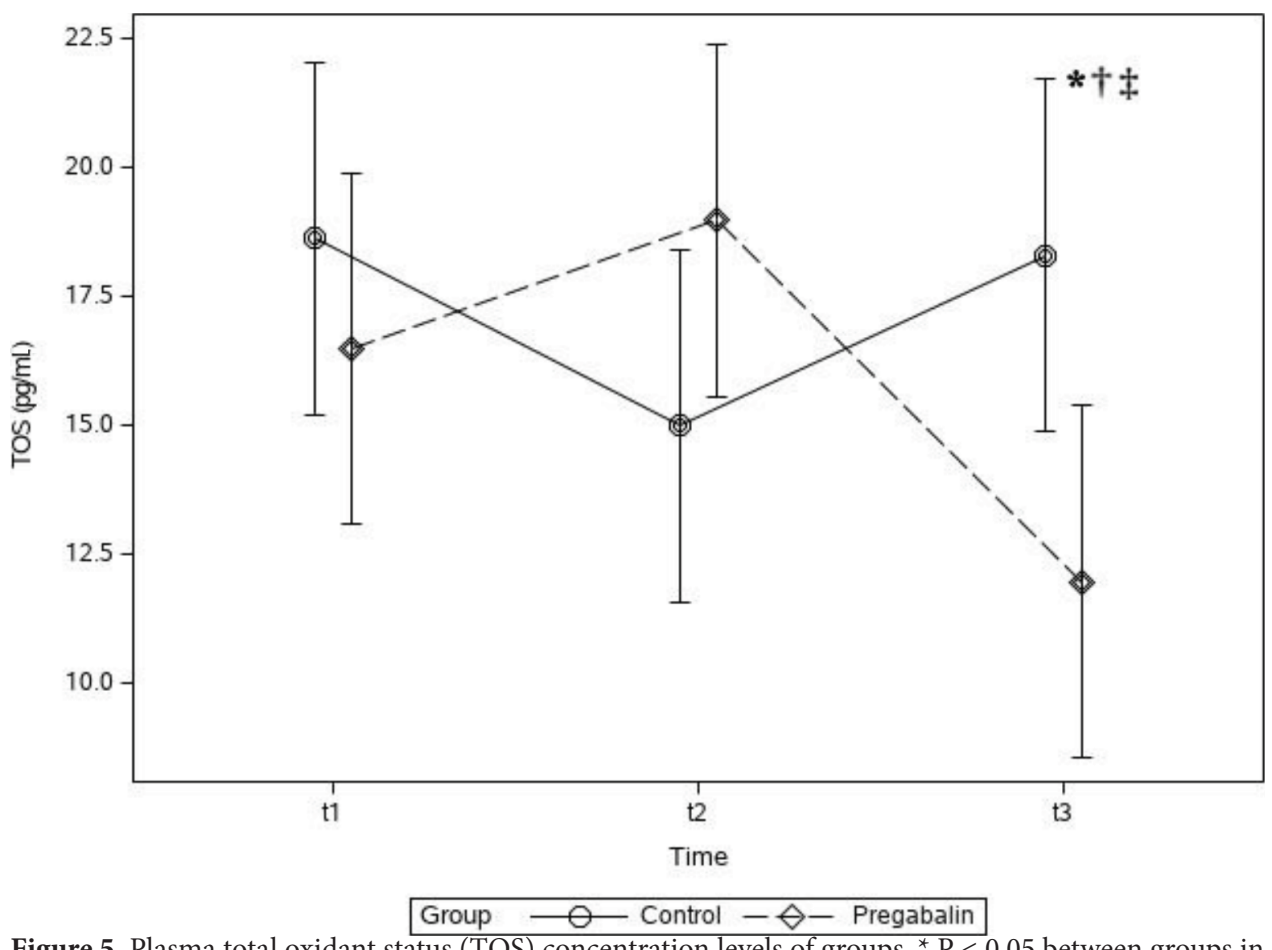
Plasma total oxidant status (TOS) concentration levels of groups. * P < 0.05 between groups in the t_3_ period. † P < 0.05 between t_3_ and t_2_ periods in the Group P. ‡ P < 0.05 between t_3_ and t_1_ periods in Group P.

Although there was no difference between the groups in terms of TOS, the group × time interaction was statistically significant (P < 0.001). In the t_3_ period, the TOS in Group P was significantly lower than that in Group C (mean ± SD, 11.97 ± 5 vs. 18.29 ± 9.9 pg/mL; P < 0.05). In Group P, the TOS was significantly lower in the t_3_ period than that in the t_2_ and t_1_ periods (mean ± SD, 11.97 ± 5 vs. 18.98 ± 10.7 pg/mL; P < 0.0001 and 11.97 ± 5 vs. 16.49; P < 0.05). In Group C, the TOS was significantly higher in the t_3_ period than that in the t_2_ period (mean ± SD, 18.29 ± 9.9 vs. 14.99 ± 6.8 pg/mL; P < 0.05) (Figure 5). 

There was no significant difference between the groups with regard to hazards ratio, SpO_2_, or mean arterial pressure during the perioperative period, or in terms of side effects of pregabalin such as confusion, dry mouth, and double or blurred vision during the perioperative and postoperative periods.

## 4. Discussion

We hypothesized that pregabalin can play a beneficial role in reducing the incidence of acute muscular ischemia and reperfusion injury caused by tourniquet application during total knee arthroplasty. The primary outcome of this study was measurement of TAS values after tourniquet release. However, there were no intergroup differences in TAS. Here, we revealed that pregabalin had no antioxidative effects against acute IRI of skeletal muscles, despite having an impact on TAS, CAT, and TOS in the early reperfusion period. Our main results were as follows: 1) the CAT level decreased in Group C, whereas it remained the same in Group P; 2) the TOS decreased in Group P, whereas it increased in Group C; and 3) in Group P, the TAS value in the reperfusion period was higher than that in the ischemia period. 

Although pregabalin is a GABA analogue, it is a potent new-generation antiepileptic agent; it exerts an effect by binding to the α2δ subunit of voltage-gated calcium channels, which causes a reduction in the Ca++ influx, and by inhibiting the release of glutamate and noradrenaline from presynaptic terminals [4,8]. 

Several previous studies demonstrated that pregabalin reduced the incidence of anxiolytic, analgesic, acute, and chronic neuropathic pain and also exerted opioid-sparing effects [15–17]. Moreover, several experimental studies reported that pregabalin decreased apoptosis and also exerted neuroprotective and antiinflammatory effects in spinal cord injury models [12,13]; furthermore, it reduced the severity of autoimmune encephalomyelitis [14], improved myelin repair, and reduced glial activation [11].

When glutamate, a Ca++-dependent excitatory neurotransmitter, enters a cell, it triggers neuronal damage and apoptosis by binding to n-methyl-d-aspartate (NMDA), which is one of the postsynaptic receptors [18–20]. Another study demonstrated that an anesthetic agent exerted protective effects against muscular IRI by inducing ischemia-reperfusion owing to its NMDA receptor-blocking property, and by reducing IRI-related lipid peroxidation markers [21]. Moreover, unlike glutamate receptor antagonists, pregabalin exhibits an excellent safety profile by suppressing Ca++ entry into presynaptic terminals and thereby reducing the release of not only glutamate but also other excitotoxic substances such as noradrenaline; thus, it shows more activity on the progressive cascade. Pregabalin also exhibits a mechanism to overcome various hazards caused by NMDA receptor antagonists [20]. 

Few studies have shown that pregabalin has protective effects against IRI, and these studies have been conducted only on animals. Asci et al. revealed that pregabalin reduced the level of lipid peroxidation markers, such as thiobarbituric acid reactive substances (TBARS), during cerebral ischemia-reperfusion, and instead increased the activity of glutathione peroxidase (SGH-PX) and CAT enzymes, which are the most important antioxidant enzymes against reactive oxygen substrates [8]. Here, pregabalin had no effect on IMA but prevented the reduction in CAT activity during the reperfusion period. 

Ozer et al. investigated the effects of different doses of pregabalin on lower limb IRI and erythrocyte deformity. Although there was no difference between the doses, they did not observe any negative effects on erythrocyte deformity in the pregabalin groups [22]. Ozturk et al. studied the effect of different doses of pregabalin (50 mg and 200 mg) on muscular ischemia-reperfusion and found that interstitial inflammation and IMA levels increased in the ischemia-reperfusion group; although the former was reduced in the pregabalin group, the drug exerted no effect on the IMA level. Additionally, the authors were of the opinion that the administration of pregabalin at high doses has a protective effect [4]. In our study, the effect of pregabalin on the level of IMA, which is produced after malondialdehyde’s interaction with plasma lipoprotein, a lipid peroxidation product, was not observed. 

The antioxidant system, which is the sum of different antioxidant products containing enzymes, nonenzymatic antioxidants, and an array of small molecules, can reduce the extent of ROS- and free radical-induced oxidative damage. These known and unknown antioxidant products form the complex antioxidant system of the human body [6]. Because TAS is a marker reflecting all antioxidant levels [23], we considered the effect on TAS value in the reperfusion period as our primary outcome. Excessive depletion of oxidants and/or antioxidants leads to the development of oxidative stress in an organism [24]. ROS produced as a result of tourniquet application-induced IRI, as well as the products formed by a series of reactions, lead to the generation of oxidative stress in an organism. Owing to these products, the injury not only causes local tissue damage but also distant organ damage [1]. CAT is one of the most important antioxidant enzymes against ROS [8]. In numerous studies, IMA levels increased in cases of acute ischemia such as that of the skeletal muscles as well as cerebral, myocardial, and pulmonary infarction/ischemia [25]. TOS is a marker reflecting the combined activity levels of all oxidants [26]. Our study is the first to investigate the protective effect of pregabalin against acute muscular IRI by demonstrating the ability of the drug to inhibit the presynaptic release of NMDA receptor-bound glutamate.  In our study, no difference was noted in the reperfusion period between Groups P and C according to the primary outcome. In contrast, we demonstrated that pregabalin reduced IRI marker levels by decreasing the TOS level; however, it increased protective factors against IRI by increasing the TAS level while maintaining the CAT level. We suggest that pregabalin is ineffective against IRI, but it contributes to the body’s antioxidant system.

This study has a few limiting factors. First, ischemia-reperfusion marker levels were not analyzed in tissues. Second, glutamate levels were not measured at the preischemic time point as well as at ischemic and reperfusion time points. Third, the actual beneficial effects on long-term variables, such as mobility of the operated knee or the first walking time during the postoperative period, were not assessed. Finally, we did not evaluate infection marker levels and immune functions. 

 In conclusion, two-time preoperative administration of 150 mg of pregabalin does not result in complete antioxidative activity. However, pregabalin contributes to the human body’s antioxidant system against oxidative stress generated by tourniquet-induced IRI occurring during total knee arthroplasty under combined spinal epidural anesthesia.

## Acknowledgment

Başkent University funding supported this study.
